# Effect of the Learning Climate of Residency Programs on Faculty’s Teaching Performance as Evaluated by Residents

**DOI:** 10.1371/journal.pone.0086512

**Published:** 2014-01-28

**Authors:** Kiki M. J. M. H. Lombarts, Maas Jan Heineman, Albert J. J. A. Scherpbier, Onyebuchi A. Arah

**Affiliations:** 1 Professional Performance Research Group, Center for Evidence-Based Education, Academic Medical Center, University of Amsterdam, Amsterdam, The Netherlands; 2 Board of Directors, Academic Medical Center, University of Amsterdam, Amsterdam, The Netherlands; 3 Office of the Dean of the Faculty of Health, Medicine and Life Sciences, University of Maastricht, Maastricht, The Netherlands; 4 Department of Epidemiology, School of Public Health, University of California Los Angeles, Los Angeles, California, United States; 5 Center for Health Policy Research, University of California Los Angeles, Los Angeles, California, United States; 6 Center for Global and Immigrant Health, Fielding School of Public Health, University of California Los Angeles, Los Angeles, California, United States; University of Westminster, United Kingdom

## Abstract

**Background:**

To understand teaching performance of individual faculty, the climate in which residents’ learning takes place, the learning climate, may be important. There is emerging evidence that specific climates do predict specific outcomes. Until now, the effect of learning climate on the performance of the individual faculty who actually do the teaching was unknown.

**Objectives:**

This study: (i) tested the hypothesis that a positive learning climate was associated with better teaching performance of individual faculty as evaluated by residents, and (ii) explored which dimensions of learning climate were associated with faculty’s teaching performance.

**Methods and Materials:**

We conducted two cross-sectional questionnaire surveys amongst residents from 45 residency training programs and multiple specialties in 17 hospitals in the Netherlands. Residents evaluated the teaching performance of individual faculty using the robust System for Evaluating Teaching Qualities (SETQ) and evaluated the learning climate of residency programs using the Dutch Residency Educational Climate Test (D-RECT). The validated D-RECT questionnaire consisted of 11 subscales of learning climate. Main outcome measure was faculty’s overall teaching (SETQ) score. We used multivariable adjusted linear mixed models to estimate the separate associations of overall learning climate and each of its subscales with faculty’s teaching performance.

**Results:**

In total 451 residents completed 3569 SETQ evaluations of 502 faculty. Residents also evaluated the learning climate of 45 residency programs in 17 hospitals in the Netherlands. Overall learning climate was positively associated with faculty’s teaching performance (regression coefficient 0.54, 95% confidence interval: 0.37 to 0.71; *P*<0.001). Three out of 11 learning climate subscales were substantially associated with better teaching performance: ‘coaching and assessment’, ‘work is adapted to residents’ competence’, and ‘formal education’.

**Conclusions:**

Individual faculty’s teaching performance evaluations are positively affected by better learning climate of residency programs.

## Introduction

The teaching performance of faculty is essential in delivering both high quality residency training and patient care. To understand teaching performance of individual faculty, the learning climate, that is the context in which residents’ learning takes place in terms of the setting, shared perceptions on policies, practices and procedures, may be important. There is emerging evidence that specific climates are important in predicting specific outcomes [1]. In health care, for example, patient safety climate has gained much attention in relation to predicting patient outcomes [2]–[4]. The focus on specific climates, such as on safety, service or learning, is a relative recent expansion of the typical conceptualization of climate as a molar or umbrella climate construct indicative of an organization’s goals and means to goal attainment [1], [4]. The specific climate constructs differ from the umbrella construct in that they examine a more narrow manifestation of the work environment than the molar climate constructs do [4]. This allows researchers to focus on more specific research goals such as measuring a patient safety climate to predict physicians’ safe behaviors. In medical education a supportive learning environment is considered to be of paramount importance to the development of trainees. Various studies have reported that learning climate affects the learners’ motivation, self-confidence and overall moral, and impacts outcomes such as academic achievements [5]–[7], student burnout [8] and medication errors [9]. Some have debated that learning climate is one of the most important factors determining the success of an effective curriculum [10]–[12]. What has not been systematically investigated up till date is the effect of learning climate on the performance of the faculty who actually do the teaching. Instead, much of the literature indicates that the significance of the environment for medical teachers is not always appreciated [11]. This is surprising because faculty, like medical trainees, also inhabit and experience the learning climate and thus are also affected by the learning environment. Moreover, we assume that the learning climate impacts on important individual level outcomes, in this case faculty’s teaching performance. This study therefore aims to explore whether a positive or supportive learning climate fosters and predicts faculty’s teaching quality. More specifically, this study has two research goals: (i) to test the hypothesis that a positive learning climate is associated with better teaching performance of faculty as evaluated by residents, and (ii) to explore which dimension(s) of learning climate is (are) associated with better teaching performance of individual faculty.

## Methods

### Study Setting and Participants

In the period September 2010 through June 2011, 451 residents from 45 residency training programs in 17 teaching hospitals in the Netherlands participated in both the System for Evaluation of Teaching Qualities (SETQ) [13]–[22] and the Dutch Residents Educational Climate Test (D-RECT) [23]–[24], evaluating individual faculty teaching performance and learning climate respectively. Evaluations were made accessible using a security code protected internet-based system and participation was anonymous. Invitations and (up till three) reminders were sent via electronic mail during the measurement period typically lasting one month for each residency program. Residents could choose to evaluate the teaching performance of many faculty, and needed to fill out only one D-RECT (learning climate) questionnaire.

### Outcome Variable: Teaching Performance

The System for Evaluation of Teaching Qualities was developed to evaluate feedback and enhance teaching performance of individual faculty. Residents and faculty (self-)evaluated faculty’s teaching performance by using an internet-based system which automatically generated and fed back to faculty individualized feedback reports covering all measured and narrated teaching qualities. The SETQ instruments were initially modeled on the Stanford Faculty Development Program (SFDP26) instrument developed in the United States [25]–[27]. We have described elsewhere the development, and properties of the specialty-specific adaption of the SETQ instruments [13]–[22]. SETQ studies have found the instruments to be reliable, valid, and feasible across specialties in (non-)academic medical centers. The SETQ system has become the number one system for evaluating individual teaching faculty in residency training in the Netherlands. Since its launch in 2008, it has been expanded to include 240 residency training programs in 52 hospitals, covering now approximately 3300 teaching faculty and 3300 residents.

Although the instruments were specialty-specific, they still shared 22 core items aimed at measuring faculty’s performance in five areas: creating a conducive learning climate (7 items), displaying a professional attitude toward residents (3 items), communicating about learning goals (4 items), evaluating residents’ knowledge and skills (4 items), and giving feedback to residents (4 items). (See Table S1.) Each item was evaluated on a 5-point Likert scale: strongly disagree, disagree, neutral, agree and strongly agree. For the statistical analyses in this study, we used the averaged overall score from all 22 items to represent each faculty’s teaching performance. This overall teaching performance score thus possible ranged from 1 to 5.

### Main Explanatory Variable: Learning Climate

Learning climate is a multifaceted concept that is complex to measure. Nevertheless, many instruments have been developed in the field of (graduate) medical educational that tap into learning climates [6], [28], [29]. In the Netherlands, the Dutch Residency Educational Climate Test (D-RECT) is the instrument used most to measure the learning climate within residency training programs as perceived by residents [23], [24]. All issues were brought to the fore by residents in earlier qualitative research and confirmed by an expert Delphi panel [23], [24]. The D-RECT instrument was found to be reliable and valid, needing only 3 to 11 resident evaluations for a reliable evaluation of each residency program’s learning climate. The instrument had 50 items with a five-point Likert response scale and was found to factor into the following 11 learning climate subscales: supervision, coaching and assessment, feedback, teamwork, peer (residents) collaboration, professional relations between attendings, work adaptation to residents’ competence levels, attendings’ attitude towards residents, formal organized education sessions, the role of the program director, and patient sign-out (Table S2). In this study, we computed the averaged composite score using all the 50 items aggregated from the residents’ evaluations up to the residency program level to represent the overall learning climate of each residency program. This possible overall learning climate score thus ranged from 1 to 5.

### Covariates

The period (month and year) of the SETQ and D-RECT measurements was used for confounding adjustment. Also, the gender and residency year of the participating residents were included as covariate adjustments.

### Statistical Analysis

Beyond the conventional descriptive analysis of our sample, we fit two types of unadjusted and adjusted random-intercepts linear mixed models to address each of our two study aims. For the first study aim of relating the outcome, teaching performance, to overall learning climate in which residents are trained, we fit linear mixed models with random intercepts for residents, faculty, residency program and hospital, without and then with adjustment for the abovementioned covariates. For the second aim, we fit similar models but replaced the overall learning climate with its 11 subscales and entered them simultaneously into the models. The random-intercepts linear mixed models with cross-classification at the level of residents and faculty were chosen to account for the crossed clustering of residents’ evaluations within residents and faculty. Accounting for this clustering using cross-classified nested random intercepts is important since different residents could evaluate different or overlapping faculty. Also, different faculty could be evaluated by different or overlapping residents, thus inducing some intra-resident and intra-faculty correlations as well as faculty-resident cross-classified heterogeneity. Similarly, we used additional random intercepts at residency program and hospital levels to further account for the hierarchical nesting of the data from faculty and residents within residency programs and, then, hospitals [30].

Associations are reported as regression coefficients with their 95% confidence interval, representing the increase (for positive coefficient) or decrease (negative coefficient) in teaching performance score given a 1-unit increase in the learning climate (or its subscale) score. We used PASW Statistics 18.0.3 for Mac (IBM SPSS Inc, 2010) for the statistical analysis.

We consulted the institutional ethical review board of the Academic Medical Center of the University of Amsterdam (AMC). They confirmed that the Medical Research Involving Human Subjects Act (WMO) did not apply to this study. We received a formal written waiver for all SETQ studies. Nevertheless, all necessary precautions were taken to guarantee and protect the anonymity and confidentiality of our study participants.

## Results

Overall, 451 residents evaluated 502 faculty on their teaching performance using the SETQ instrument. A total of 3,569 SETQ evaluations were completed by the residents in 45 residency programs representing 18 medical and surgical specialties in 17 hospitals in the Netherlands (Table 1). In addition, a mean of 6 residents and a median of 7 (inter-quartile range 4–10) residents evaluated the learning climate of the same 45 residency training programs.

**Table 1:** Characteristics of the study sample.

Characteristic	N or mean (SD)
Number of faculty whose teaching performance were evaluated	502
Number of residents who evaluated faculty’s teaching performance	451
Number of faculty’s teaching performance evaluations	3569
Number of medical or surgical specialties	18
Number of hospitals	17
Number of residency programs	45
Overall teaching performance score: mean (SD)	3.78 (0.62)
Overall learning climate score: mean (SD)	3.71 (0.33)
Learning climate subscale scores	
• Supervision: mean (SD)	3.93 (0.36)
• Coaching and assessment: mean (SD)	3.24 (0.35)
• Feedback: mean (SD)	3.24 (0.45)
• Teamwork: mean (SD)	3.59 (0.51)
• Peer collaboration: mean (SD)	4.28 (0.28)
• Professional relations between attendings: mean (SD)	3.61 (0.67)
• Work is adapted to residents’ competence: mean (SD)	3.67 (0.39)
• Attendings’ role: mean (SD)	3.94 (0.34)
• Formal education: mean (SD)	3.68 (0.42)
• Role of the specialty tutor: mean (SD)	3.98 (0.51)
• Patient sign-out: mean (SD)	3.82 (0.46)

SD: standard deviation.

Table 2 shows the results of the analysis of the first study aim. We found that overall learning climate of the residency programs was positively associated with faculty’s teaching performance. In the multivariable adjusted linear model, the regression coefficient for the overall learning climate was 0.54 with a 95% confidence interval of 0.37 to 0.71 (*P*<0.001). Table 3 shows the results for the second aim that focused on the learning climate subscales. Of the 11 learning climate subscales, three appeared to be substantially associated with better teaching performance: ‘coaching and assessment’ (0.41; 95% CI: 0.19 to 62; *P*<0.001), ‘work adaptation to residents’ competence levels’ (0.37; 95% CI: 0.05 to 69; *P* = 0.023), and ‘formal education sessions’ (0.16; 95% CI: 0.01 to 0.32; *P* = 0.04). Teamwork appeared to be somewhat negatively associated with teaching performance although the confidence interval crossed the null (−0.13; 95% CI: −0.39 to 0.02; *P* = 0.082).

**Table 2:** Unadjusted and adjusted associations of overall learning climate score with overall teaching performance of faculty as evaluated by residents.

	Unadjusted faculty’s teaching performance (overall scale; unadjusted for covariates)			Adjusted faculty’s teaching performance (overall scale; adjusted for covariates^a^)		
Learning climate of residency programs	Regression coefficient	95% Confidence interval	*P* value	Regression coefficient	95% Confidence interval	*P* value
Overall mean score for learning climate	0.45	0.32 to 0.59	<0.001	0.54	0.37 to 0.71	<0.001

a Adjusted for evaluating resident’s gender and residency year, and evaluation period in cross-classified random-intercept linear mixed regression.

**Table 3:** Unadjusted and adjusted joint associations of learning climate subscales with overall teaching performance of faculty as evaluated by residents.

	Unadjusted faculty’s teaching performance (overall scale; unadjusted for covariates)			Adjusted faculty’s teaching performance (overall scale; adjusted for covariates^a^)		
Learning climate of residency programs	Regression coefficient	95% Confidence interval	*P* value	Regression coefficient	95% Confidence interval	*P* value
Supervision	−0.10	−0.26 to 0.07	0.244	−0.14	−0.33 to 0.04	0.134
Coaching and assessment	0.34	0.18 to 0.50	<0.001	0.41	0.19 to 0.62	<0.001
Feedback	0.05	−0.04 to 0.14	0.310	0.10	−0.03 to 0.23	0.125
Teamwork	−0.13	−0.25 to 0.00	0.054	−0.18	−0.39 to 0.02	0.082
Peer (residents)collaboration	0.08	−0.09 to 0.26	0.337	0.13	−0.11 to 0.36	0.277
Professional relations between attendings	0.04	−0.08 to 0.16	0.507	0.00	−0.18 to 0.17	0.963
Work is adapted toresidents’ competence	0.17	−0.03 to 0.38	0.096	0.37	0.05 to 0.69	0.023
Attendings’ role	−0.01	−0.25 to 0.23	0.939	−0.06	−0.35 to 0.23	0.695
Formal education	0.15	0.03 to 0.28	0.017	0.16	0.01 to 0.32	0.040
Role of the programdirector	−0.01	−0.14 to 0.12	0.884	−0.07	−0.23 to 0.08	0.349
Patient sign-out	−0.04	−0.18 to 0.11	0.625	−0.11	−0.30 to 0.09	0.275

a All models adjusted for evaluating resident’s gender and residency year, and evaluation period in cross-classified random-intercept linear mixed regressions.

## Discussion

### Main Findings

This study provides strong empirical evidence that faculty’s teaching performance is positively affected by the learning climate of the residency training program. Of the eleven predefined learning climate dimensions three appear to most convincingly predict teaching performance: ‘coaching and assessment’, ‘work adaptation to residents’ competence levels’ and ‘formal organized education’.

### Strengths and Limitations of the Study

This paper adds to the understanding of the teaching performance of clinicians and the potential improvement of clinical teaching. To our knowledge this study is the first multi-center study to assess the associations of the learning climate’s overall scale and subscales with teaching performance of individual faculty in a diverse sample of residents and faculty. We were able to use two validated and widely known and accepted instruments for measuring the learning climate (D-RECT) in residency training and faculty’s individual teaching performance (SETQ). The response rates for both instruments were high, likely due to the anonymous participation of residents in the evaluations, the web- based user-friendly data collection as well as the frequent communication between (one of) the researchers and program directors about the progress development of response rates. This study was limited by its cross-sectional design, which precludes determination of whether the overall learning climate or separate dimensions of the learning environment are causally related to teaching performance.

### Explanation of the Findings

In medical education, a supportive learning climate is considered to be of paramount importance to the development of knowledge and skills of residents [11], [12], [28]. This study reports that a learning climate that is viewed as beneficial by residents is predictive of better performance of teaching faculty as perceived by the same residents. Unsurprisingly, the learning climate dimensions showing the strongest associations with teaching performance are those who reflect most clearly the direct learning interactions between teachers and residents, namely, coaching of residents, organized formal education sessions and adaption of the work to the resident’s competence level. Other dimensions (see Table S2) may be more conditional for these learning interactions, such as the organization of supervision of residents, the role of the program director or the collaboration amongst peer residents. In interpreting our findings we should be cautious assuming causal interpretations between the learning climate and teaching performance of faculty. However, both teaching performance and learning climate can be expected to affect each other in most settings. Climate researchers in the field of human resource management have developed a few path models in an attempt to explain the link between climate and outcomes [4], [31]. Some argued and found evidence for the idea that a *general* climate factor accounts for the relationship between climate dimensions and outcomes [31]. Others, however, found that different climate dimensions interact differently with (intermediate) outcomes [4]. Our study underpins the idea of specific learning climates affecting individual outcomes.

### Implications for Clinical Education, Research and Policy

It has been pointed out that the creation of an environment that is conducive to teaching is not given much attention [11], [12]. In the interest of providing high quality training for residents it can be recommended, based on the findings presented in this study, that teaching institutions facilitate in the provision of the most appropriate climate in which teachers (and residents alike) operate on a day-to-day basis. The good news is that there is evidence that climate can be changed [11], [12]. Future research will need to further investigate which factors mediate the relationship between learning climate and faculty’s teaching performance.

## Conclusions

Individual faculty’s teaching performance evaluations are positively affected by better learning climates in residency programs. This finding is likely due to those learning climate aspects that are more proximal to faculty-resident learning interactions. This understanding may be instrumental for institutions in further improving the quality of residency training.

## Supporting Information

Table S1 The 5 subscales and 22 items that overlap on the SETQ instruments.
(DOC)

Table S2 The 11 subscales and corresponding 50 items of the D-RECT instrument.
(DOC)

## References

[1] Patterson M, Rick J, Wood S, Carroll C, Balain S, et al.. (2010) A Systematic review of the links between human resource management practices and performance. Health Technol Assess 14: 1–334, iv. 10.3310/hta14510 21050585

[2] Flin R, Burns C, Mearns K, Yule S, Robertson EM (2006) Measuring safety climate in health care. Qual Saf Health Care 15: 109–15. 16585110 10.1136/qshc.2005.014761 PMC2464831

[3] Colla JB, Bracken AC, Kinney LM, Weeks WB (2005) Measuring patient safety climate: a review of surveys. Qual Saf Health Care 14: 364–6. 16195571 10.1136/qshc.2005.014217 PMC1744072

[4] Carr JZ, Schmidt AM, Ford JK, DeShon RP (2003) Climate perceptions matter: a meta-analytic path analysis relating molar climate, cognitive and affective states, and individual level work outcomes. J Appl Psychol 88: 605–19. 12940402 10.1037/0021-9010.88.4.605

[5] Abraham R, Ramnarayan K, Vinod P, Torke S (2008) Students perception of learning environment in an Indian Medical School. BMC Med Educ 8: 20 doi: 10.1186/1472-6920-8-20 18402710 10.1186/1472-6920-8-20 PMC2329622

[6] Saito A, Sunell S, Rucker L, Wilson M, Sato Y, et al (2010) Learning climate in dental hygiene education: a longitudinal case study of a Japanese and Canadian programme. Int J Dent Hyg 8: 134–42. 20522137 10.1111/j.1601-5037.2009.00411.x

[7] Lucas CA, Benedek D, Pangaro L (1993) Learning climate and students’ achievement in a medicine clerkship. Acad Med 68: 811–2. 10.1097/00001888-199310000-00025 8397620

[8] Dyrbye LN, Thomas MR, Harper W, Massie FS, Power DV, et al (2009) The learning environment and medical student burnout: a multicentre study. Med Educ 43: 274–82. 19250355 10.1111/j.1365-2923.2008.03282.x

[9] Chang Y, Mark B (2011) Effects of learning climate and registered nurse staffing on medication errors. Nurs Res 60: 32–9. 21127452 10.1097/NNR.0b013e3181ff73cc PMC3086538

[10] Bassaw B, Roff S, McAleer S, Roopnarinesing S, Lisle JD, et al (2003) Students’ perspectives on the educational environment, Faculty of Medical Sciences, Trinidad. Med Teach 25: 522–6. 14522676 10.1080/0142159031000137409

[11] Genn JM (2001) AMEE Medical Education Guide No 23 (Part 1): Curriculum, environment, climate, quality and change in medical education–a unifying perspective. Med Teach 23: 337–44. 12098379 10.1080/01421590120063330

[12] Genn JM (2001) AMEE Medical Education Guide No. 23 (Part 2): Curriculum, environment, climate, quality and change in medical education - a unifying perspective. Med Teach 23: 445–454. 12098364 10.1080/01421590120075661

[13] Lombarts MJ, Bucx MJ, Rupp I, Keijzers PJ, Kokke SI, et al.. (2007) An instrument for the assessment of the training qualities of clinician-educators. Ned Tijdschr Geneeskd 151: 2004–8. [in Dutch]. 17953176

[14] Lombarts MJ, Bucx MJ, Arah OA (2009) Development of a system for the evaluation of the teaching qualities of anesthesiology faculty. Anesthesiology 111: 706–19. 19707115 10.1097/ALN.0b013e3181b76516

[15] Lombarts MJMH, Busch ORC, Heineman MJ, Arah O (2010) Ned Tijdschr Geneeskd 154: A1222. [in Dutch]. 20170574

[16] Lombarts KM, Heineman MJ, Arah OA (2010) Good clinical teachers likely to be specialist role models: results from a multicenter cross-sectional survey. PLoS One. 2010 5(12): e15202. 10.1371/journal.pone.0015202 PMC3012058 21206906

[17] Van der Leeuw R, Lombarts K, Heineman MJ, Arah O (2011) Systematic evaluation of the teaching qualities of Obstetrics and Gynecology faculty: reliability and validity of the SETQ tools. PLoS One 6: e19142. 21559275 10.1371/journal.pone.0019142 PMC3086887

[18] Arah OA, Hoekstra JBL, Bos AH, Lombarts MJMH (2011) New Tools for Systematic Evaluation of Teaching Qualities of Medical Faculty: Results of an Ongoing Multi-Center Survey. PLoS One 6: e25983. 22022486 10.1371/journal.pone.0025983 PMC3193529

[19] Arah OA, Heineman MJ, Lombarts KM (2012) Factors influencing residents’ evaluations of clinical faculty member teaching qualities and role model status. Med Educ 46(4): 381–9. 22429174 10.1111/j.1365-2923.2011.04176.x

[20] Boerebach BC, Arah OA, Busch OR, Lombarts KM (2012) Reliable and valid tools for measuring surgeons’ teaching performance: residents’ vs. self evaluation. J Surg Educ 69511–20. 10.1016/j.jsurg.2012.04.003 22677591

[21] Boerebach BC, Lombarts KM, Keijzer C, Heineman MJ, Arah OA (2012) The teacher, the physician and the person: how faculty’s teaching performance influences their role modelling. PLoS One 7(3): e32089. 22427818 10.1371/journal.pone.0032089 PMC3299651

[22] Boerebach BC, Lombarts KM, Scherpbier AJ, Arah OA (2013) The teacher, the physician and the person: exploring causal connections between teaching performance and role model types using directed acyclic graphs. PLoS One 2013 8(7): e69449. 10.1371/journal.pone.0069449 PMC3720648 23936020

[23] Boor K, Van Der Vleuten C, Teunissen P, Scherpbier A, Scheele F (2011) Development and analysis of D-RECT, an instrument measuring residents learning climate. Med Teach 33: 820–827. 21355691 10.3109/0142159X.2010.541533

[24] Boor K (2009) The clinical learning climate. Dissertation. Amsterdam: VU Medical Center.

[25] Skeff KM, Stratos GA, Berman J, Bergen MR (1992) Improving clinical teaching. Evaluation of a national dissemination program. Arch Intern Med 152: 1156–61. 1599342 10.1001/archinte.152.6.1156

[26] Litzelman DK, Stratos GA, Marriott DJ, Skeff KM (1998) Factorial validation of a widely disseminated educational framework for evaluating clinical teachers. Acad Med 73: 688–95. 9653408 10.1097/00001888-199806000-00016

[27] Williams BC, Litzelman DK, Babbott SF, Lubitz RM, Hofer TP (2002) Validation of a global measure of faculty’s clinical teaching performance. Acad Med 77: 177–80. 11841985 10.1097/00001888-200202000-00020

[28] Roff S (2005) Education environment: a bibliography. Med Teach 27: 353–7. 16024420 10.1080/01421590500151039

[29] Kanashiro J, McAleer S, Roff S (2006) Assessing the educational environment in the operating room–a measure of resident perception at one Canadian institution. Surgery 139: 150–158. 16455322 10.1016/j.surg.2005.07.005

[30] Gelman A, Hill J (2007) Data Analysis Using Regression and Multilevel/Hierarchical models. Cambridge: Cambridge University Press.

[31] Parker CP, Baltes BB, Huff JW, Altmann RA, LaCost HA, et al (2004) Relationships between Psychological Climate Perceptions and Work Outcomes: A Meta-Analytic Review. J Org Beh 24: 389–416.

