# Supplementary material for: Effect of the Learning Climate of Residency Programs on Faculty’s Teaching Performance as Evaluated by Residents
Source: PLoS One. 2014 Jan 28;9(1):e86512. doi: 10.1371/journal.pone.0086512 (PMC3904911; doi:10.1371/journal.pone.0086512)
Supplement: Table S1 — The 5 subscales and 22 items that overlap on the SETQ instruments. (DOC) [file pone.0086512.s001.doc]

Table A1: The 5 subscales and 22 items that overlap on the SETQ instruments

| **Item number** | **Scale**  **and items** |
| --- | --- |
|  | *Learning climate* |
| L1 | Encourages residents to participate actively in discussions |
| L2 | Stimulates residents to bring up problems |
| L3 | Teaches residents time management |
| L4 | Keeps to teaching goals; avoids digressions |
| L5 | Motivates residents to study further |
| L6 | Stimulates residents to keep up with the literature |
| L7 | Prepares well for teaching presentations and talks |
|  | *Professional attitude towards residents* |
| P1 | Listens attentively to residents |
| P2 | Is respectful towards residents |
| P3 | Is easily approachable during on-calls |
|  | *Communication of goals* |
| C1 | States learning goals clearly |
| C2 | States relevant goals |
| C3 | Prioritizes learning goals |
| C4 | Repeats stated learning goals periodically |
|  | *Evaluation of residents’ knowledge and skills* |
| E1 | Evaluates residents’ specialty knowledge regularly |
| E2 | Evaluates residents’ analytical abilities regularly |
| E3 | Evaluates residents’ application of knowledge to specific patients regularly |
| E4 | Evaluates residents’ medical skills regularly |
|  | *Feedback* |
| F1 | Regularly gives positive feedback to residents |
| F2 | Gives corrective feedback to residents |
| F3 | Explains why residents are incorrect |
| F4 | Offers suggestions for improvement |
