# Supplementary material for: Effect of the Learning Climate of Residency Programs on Faculty’s Teaching Performance as Evaluated by Residents
Source: PLoS One. 2014 Jan 28;9(1):e86512. doi: 10.1371/journal.pone.0086512 (PMC3904911; doi:10.1371/journal.pone.0086512)
Supplement: Table S2 — The 11 subscales and corresponding 50 items of the D-RECT instrument. (DOC) [file pone.0086512.s002.doc]

Table A2: The 11 subscales and corresponding 50 items of the D-RECT instrument

| *Supervision* |
| --- |
| 1. The guidelines clearly outline when to request input from a supervisor |
| 1. The amount of supervision I receive is appropriate for my level of experience |
| 1. It is clear which attending supervises me |
| *Coaching and assessment* |
| 1. I am asked on a regular basis to provide a rationale for my management decisions and actions |
| 1. My attendings coach me on how to communicate with difficult patients |
| 1. My attendings take the initiative to explain their actions |
| 1. My attendings take the initiative to evaluate my performance |
| 1. My attendings take the initiative to evaluate difficult situations I have been involved in |
| 1. My attendings evaluate whether my performance in patient care is commensurate with my level of training |
| 1. My attendings occasionally observe me taking a history |
| 1. My attendings assess not only my medical expertise but also other skills such as teamwork, organization or professional behavior |
| *Feedback* |
| 1. My attendings give regular feedback on my strengths and weaknesses |
| 1. Observation forms (i.e., Mini-CEX) are used to structure feedback |
| 1. Observation forms (i.e., Mini-CEX) are used periodically to monitor my progress |
| *Teamwork* |
| 1. Attendings, nursing staff, other allied health professionals and residents work together as a team |
| 1. Nursing staff and other allied health professionals make a positive contribution to my training |
| 1. Nursing staff and other allied health professionals are willing to reflect with me on the delivery of patient care |
| 1. Teamwork is an integral part of my training |
| *Peer collaboration* |
| 1. Residents work well together |
| 1. Residents, as a group, make sure the day’s work gets done |
| 1. Within our group of residents it is easy to find someone to cover or exchange a call |
| *Professional relations between attendings* |
| 1. Continuity of care is not affected by differences of opinion between attendings |
| 1. Differences of opinion between attendings about patient management are discussed in such a manner that is instructive to others present |
| 1. Differences of opinion are not such that they have a negative impact on the work climate |
| *Work is adapted to residents’ competence* |
| 1. The work I am doing is commensurate with my level of experience |
| 1. The work I am doing suits my learning objectives at this stage of my training |
| 1. It is possible to do follow up with patients |
| 1. There is enough time in the schedule for me to learn new skills |
| *Attendings’ role* |
| 1. My attendings take time to explain things when asked for advice |
| 1. My attendings are happy to discuss patient care |
| 1. There is (are) NO attending physician(s) who have a negative impact on the educational climate |
| 1. My attendings treat me as an individual |
| 1. My attendings treat me with respect |
| 1. My attendings are all in their own way positive role models |
| 1. When I need a attending, I can always contact one |
| 1. When I need to consult an attending, they are readily available |
| *Formal education* |
| 1. Residents are generally able to attend scheduled educational activities |
| 1. Educational activities take place as scheduled |
| 1. Attendings contribute actively to the delivery of high-quality formal education |
| 1. Formal education and training activities are appropriate to my needs |
| *Role of the specialty tutor* |
| 1. The specialty tutor monitors the progress of my training |
| 1. The specialty tutor provides guidance to other attendings when needed |
| 1. The specialty tutor is actively involved in improving the quality of education and training |
| 1. In this rotation evaluations are useful discussions about my performance |
| 1. My plans for the future are part of the discussion |
| 1. During evaluations, input from several attendings is considered |
| *Patient sign-out* |
| 1. When there is criticism of a management plan I have developed in consultation with my attending physician, I know the attending physician will back me up |
| 1. Sign out takes place in a safe climate |
| 1. Sign out is used as a teaching opportunity |
| 1. Attendings encourage residents to join in the discussion during sign out |
